# Donation, Not Disease! A Multiple-Hit Hypothesis on Development of Post-Donation Kidney Disease

**DOI:** 10.1007/s40472-017-0171-8

**Published:** 2017-11-04

**Authors:** Xingxing S. Cheng, Richard J. Glassock, Krista L. Lentine, Glenn M. Chertow, Jane C. Tan

**Affiliations:** 1 Division of Nephrology, Department of Medicine, Stanford University, 750 Welch Road, Suite 200, Mail code 5785, Palo Alto, CA 94304 USA; 2 Department of Medicine, David Geffen School of Medicine at UCLA, Los Angeles, CA USA; 3 Division of Nephrology, Saint Louis University Center for Abdominal Transplantation, Saint Louis, MO USA

**Keywords:** Living kidney donation, Post-donation kidney disease, Risk assessment

## Abstract

**Purpose of Review:**

The risks following living kidney donation has been the subject of rigorous investigation in the past several decades. How to utilize the burgeoning new knowledge base to better the risk assessment, education, and health maintenance of donors is unclear. We review the physiologic and epidemiologic evidences on the post-donation state and submit a multiple-hit hypothesis to reconcile the finite elevation in risk of kidney disease after donation with the benign course of most kidney donors.

**Recent Findings:**

The risk of end-stage kidney disease is higher in kidney donors compared to similarly healthy non-kidney donors. Nonetheless, post-donation kidney disease is uncommon and arises mostly in the setting of other “hits”—either a “first hit” present at birth or a “second hit” acquired later in life.

**Summary:**

The transplant community’s focus should be directed toward (1) personalized risk assessment to inform consent before donation and (2) preventing and treating development of “second hits” following kidney donation.

## Introduction

Each year, more than 60,000 living kidney donations occur worldwide. Kidney donation entails surgically removing approximately 50% of the functional nephron mass from an apparently healthy donor. Previously thought to have trivial medical risks, living kidney donation has been subject to increasingly rigorous investigations that have raised concern over the long-term medical risks of donation [1]. As physicians, sworn to uphold both tenets of “do no harm” to potential donors and “do good” to patients in need of a transplant, how are we to proceed with this new knowledge?

A reasonable approach is fully informed consent, comprehensively counseling patients on their individual risks and benefits associated with donation so they can make a truly informed decision. However, it is difficult to assess risk accurately on an individual basis. Because each individual’s risk of post-donation kidney disease is very low, detection of an elevated risk necessitates pooling effect sizes over large patient cohorts, and the proper interpretation of signals obtained in this setting requires extra care.

We contend that the elevated risk of progressive post-donation kidney disease seen in large, epidemiologic cohorts is not a uniform risk applicable to all living donors, but rather a function of donor subsets at variable levels of risk. In other words, donation per se is probably not sufficient to cause clinically meaningful chronic kidney disease (CKD) in isolation, other factors (first and second “hits”) are at play, and our understanding of these factors is still incomplete. We arrive at this conclusion through careful integration of clinical and physiologic studies, not only of kidney donors, but also of CKD progression in non-donors. In this perspectives paper, we review some of the existing evidence on physiologic changes post-donation, put forth our model, and discuss ways in which this model may assist in better donor education, assessment, and management.

### All Kidney Donors Undergo Nephron Mass Reduction, Adaptive Hyperfiltration of Residual Nephrons and a Moderate Decrement in Whole-Kidney Glomerular Filtration Rate

In the 1970s, Brenner et al. established, via an experimental rat model, the causal relation among significantly decreased nephron mass (5/6-nephrectomy), single-nephron hyperfiltration due to glomerular hypertension and hyper-perfusion, and structural lesions of kidney disease [2]. The 5/6-nephrectomy model has formed the cornerstone of our understanding of kidney disease pathogenesis and progression, as well as highlighted the crucial link between nephron mass and kidney function. Healthy young adults are typically endowed with approximately 980,000 functioning nephrons per kidney, with very large inter-individual variation (range 200,000 to 1,800,000 nephrons per kidney) [3, 4]. Despite this wide variation in nephron number, the whole-kidney glomerular filtration rate (GFR) is much less variable within the healthy population, indicating a compensatory change in single-nephron GFR in individuals with lower nephron number at birth. With normal aging, the number of non-sclerotic, functioning nephrons declines such that about 50% of the initial nephron mass remains by age 70–75 years. The main source of variation in initial nephron number appears to be differential nephrogenesis in utero [5]. Because nephron number cannot be clinically assessed, multiple clinical parameters—including birth weight, preterm birth, adult height, and kidney volume—have been used as surrogates and have been associated with hypertension and kidney disease at a population level [6].

What changes vis-à-vis kidney functions after donor nephrectomy? While nephron mass is halved at the time of the operation, GFR in the remaining kidney increases by approximately 20–40% by 1–2 weeks post-donation, reflecting an increase in the single-nephron GFR by the remaining nephrons [7, 8]. Despite the presumed steady loss on nephrons from aging, the positive slope for post-donation GFR continues through the first several years post-donation [9•, 10•] and probably even beyond [11•], suggesting ongoing compensation within the first decade of donation. Thus is compensatory hyperfiltration inevitable after kidney donation. Whether hyperfiltration is adaptive (benign) or maladaptive (pathogenic) depends on multiple physiologic factors. Physiologic studies using mathematical modeling in human donors after nephrectomy tend to refute a role for glomerular hypertension and have implicated a possible increase in filtration surface area or glomerular plasma flow as responsible physiologic mediators [8, 9•]. These latter mechanisms are generally considered benign, but some donors may be particularly vulnerable. We postulate that donors with preexisting low nephron endowment from impaired nephrogenesis in utero may be the most vulnerable to glomerular hypertension and progressive chronic kidney disease (CKD) in a manner similar to the 5/6-nephrectomy rat model. This thesis, first advanced by Brenner and colleagues in 1988, implicated nephron endowment as a determinant of essential hypertension [12] and was later extended to involve many forms of CKD [5, 6, 13]. In this context, it is noteworthy that the development of systemic arterial hypertension is quite common among transplant donors [7]. To support the notion that pathologic hyperfiltration is the exception, rather than the rule, we note that most donors do not develop progressive, proteinuric kidney disease after donation, as would be expected if post-donation hyperfiltration accompanied by glomerular capillary hypertension were invariably pathologic.

**Fig. 1:**
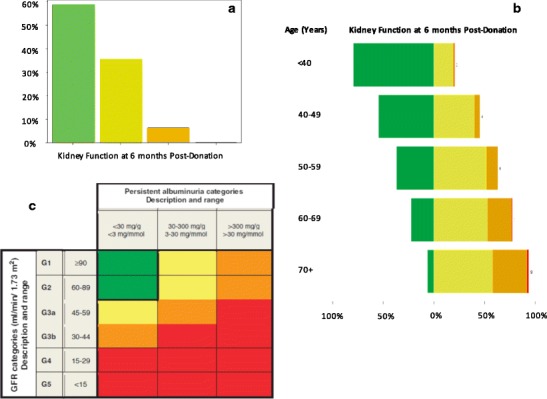
Application of estimated glomerular filtration rate (eGFR)-based chronic kidney disease (CKD) classification to the 6-month serum-creatinine derived eGFR in 25,595 living kidney donors in Scientific Registry Transplant Recipients, without (**a**) and with (**b**) stratification by age at time of donation. The Kidney Disease Improving Global Outcomes classification 2012 is included (**c**) for reference

Despite the generally benign post-donation course in most living kidney donors, many donors do have a sustained, mild decrement in GFR after donation compared to their pre-donation GFR. The degree may be overstated by the most commonly used methods of assessment, estimating equations based on serum creatinine, which have been uniformly validated in binephric cohorts [14, 15] (Fig. 1). However, even where GFR is measured (by iothalamate clearance), up to 36% of donors have measured GFR < 60 ml/min/1.73m^2^ meeting the Kidney Disease Improving Global Outcomes (KDIGO) definition for stage 3 CKD after a mean follow-up of 18 years [11•, 15, 16]. It is not yet clear whether a further decline in GFR occurs after additional decades of follow-up.

Possibly related to the decrement in GFR, post-donation arterial hypertension is reported in roughly one in three of predominantly white donors in the first 14 years after donation [11•]. The risk of post-donation hypertension is further elevated by 37% in African-American donors relative to Caucasian donors [17]. We speculate that the heightened risk of post-donation hypertension in African-Americans may be a manifestation of the connections among nephron endowment, salt-sensitivity and hypertension [12], as well as the risks of progressive glomerulosclerosis in African-Americans who possess two high-risk variants at the ApoL1 gene locus [18]. Alternatively, the putative factors driving compensatory renal growth after donation may themselves modulate blood pressure.

Recent research has drawn attention to the more subtle extra-renal manifestations possibly related to the decrement in GFR post-donation, which include an increased relative risk of gestational hypertension [19] and gout [20] compared to matched controls. The overall cardiovascular impact of the modest decline in whole-kidney GFR in an otherwise healthy adult is uncertain, but some prospective controlled studies have suggested that such alterations might have an effect on left ventricular mass even independent of blood pressure in otherwise healthy donors [18, 19]. Other asymptomatic biochemical abnormalities, including elevations in fibroblast growth factor 23 and other biomarkers of mineral and bone metabolism, have also be observed [21].

In summary, we concur with the views of the transplant community that the physiologic compensation after kidney donation seems to be benign for most donors. A modest decrement in GFR and a greater propensity toward arterial hypertension and albuminuria are the main adverse effects of donation. In addition, subtle alterations in cardiac structure, urate excretion, and bone and mineral metabolism arise, the clinical significance of which remain undefined.

### Development of Post-Donation Kidney Disease: A Multiple-Hit Process

**Fig. 2:**
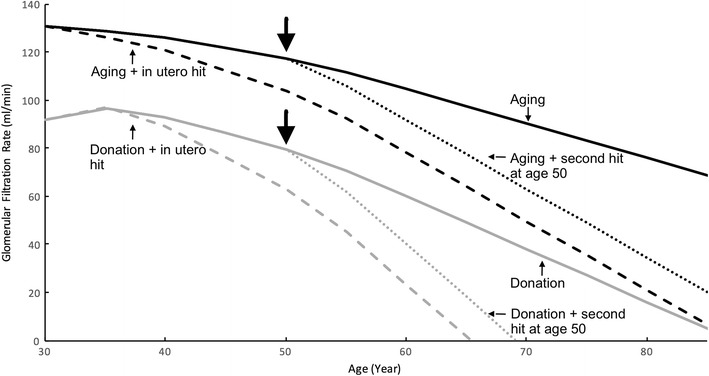
A schematic presentation of the multiple-hit hypothesis of kidney disease progression in living donors. A hypothetical 30-year-old candidate contemplates kidney donation with three possible scenarios: (1) without donation and with perfect health, glomerular filtration rate (GFR) declines gradually with age (black solid line, adopted from []). With donation, adaptive compensation keeps GFR at ~ 70% of binephric level (gray solid line). (2) A “first hit” exists (in utero insult or genetic predisposition), and GFR declines at 2× the normal rate, with and without donation (hashed lines). (3) A medical risk factor develops at age 50 and causes GFR to decline at 2X the normal rate, with and without donation (dotted lines). In scenarios 2) and 3), the donor’s time to ESKD is significant shortened by donation

Brenner et al. have long hypothesized that inadequate nephron endowment at birth partially explains disparate rates of kidney disease progression in the general, binephric CKD population [12, 22]. Based on these observations and the physiologic principles described above, we suggest application of a “multiple-hit” process of kidney disease progression in donors as an extension of the Brenner postulates linking nephron endowment to CKD (Fig. 2]).

In a healthy, aging person without an in utero insult or genetic predisposition, GFR decreases in parallel with renal blood flow [23], histologically correlated with a reduced number of functioning glomeruli [4, 24]. In a donor, GFR increases somewhat in the first decade as a result of hyperfiltration [10•, 25, 26], despite the fact that nephron number gradually declines with age [4]. The point at which hyperfiltration might transform from a benign adaptive process to a pathogenic process is unknown, and likely varies widely from person to person. Kidney donation shortens time to end-stage kidney disease (ESKD) in two scenarios. In the presence of a “first hit” present at birth, such as low nephron endowment or genetic predisposition, post-donation physiologic adaptations can lead to glomerular hypertension and accelerate age-related GFR decline [5, 12]. Alternatively, with the acquisition of a “second hit” later in life, e.g., diabetes mellitus, obesity, and/or arterial hypertension, donors will have diminished nephron reserve with which to withstand the additional impact of the new pathologic process, and time to ESKD may be shortened [11•, 27].

Of note, past and current transplant practices seldom assess potential donors for presence of the “first hit,” and most studies reporting long-term renal outcomes in donors have incomplete information on the acquisition of the “second hit.” Thus, this hypothesis is hitherto untested, to the best of our knowledge.

How does this framework alter our interpretation of the literature to date? The incidence of post-donation ESKD was reported to be 0.3 per 1000-person-years in a Norwegian national cohort [28] and was 3.1 per 1000-persons at 15 years in a US national cohort [29•], reflecting a tenfold higher relative risk compared to healthy non-donors, although the absolute lifetime risk of ESKD attributable to donation was low. We would suggest that the elevated risk of ESKD post-donation is not uniformly distributed, but rather concentrated in subsets of donors who have a “first hit” or who will acquire an additional “second hit.” For instance, in the Minnesota cohort, the best-characterized long-term donor follow-up study to date, 6.1% developed albuminuria after a median follow-up of 17 years [11•]. Albuminuria is long established as among the earliest manifestations of kidney disease [22]. In this study, albuminuria was independently associated with death and kidney failure. We surmise that those donors who develop albuminuria early post-donation probably had a “first hit” that was not appreciated at the time of kidney donation; in other words, early albuminuria post-donation may be a marker of an unrecognized predisposition and signals a donor at high risk of CKD progression and possibly cardiovascular adverse events. In a second subset of patients, a “second-hit” (e.g., diabetes mellitus, reported in 5–6% of donors within the first two decades of donation [11•, 17], or obesity [27]) develops in the years or decades after donation, and these may account for the late post-donation CKD cases. Corroborating this, a recent analysis of causes of ESKD in US donors highlighted the fact that most post-donation ESKD cases arising beyond the first decade after donation are attributed to hypertensive and diabetic kidney disease [30]. Together, these two donor subsets likely account for most of the elevated ESKD risk in donors.

Recent breakthroughs have identified novel candidates for the “first hit” in our hypothesis as proposed above. These include the following:

1. *APOL1* genotype: In 2010, two landmark papers reported that high-risk APOL1 variants are highly prevalent in the African American population (13%) and confer a 15% lifetime risk of CKD [31]. This dovetails with the observation that African American donors also have a higher risk of developing ESKD [29•]. Whether this elevated risk is mostly attributable to high-risk APOL1 variants is unknown, and whether the degree of risk elevation is enough to justify excluding the donor candidate from donation is hotly debated. The latest consensus document from the American Society of Transplantation recommends informing all African-American donor candidates about the association between high-risk APOL1 alleles and kidney disease even in the absence of donation and offering genetic testing as a part of the donor evaluation, but concludes that insufficient evidence is available to recommend testing all African-American donor candidates or to determine acceptance or exclusion of donor candidates based on APOL1 genotype alone [31]. The quandary of the presence of high-risk APOL1 variants in potential donors is a “known unknown” soon to be elucidated by national research efforts [32].
2. Low birth weight: Low birth weight is a useful surrogate for low nephron endowment. In a cohort of 91 Caucasian donors from Germany [33], stratified into groups by birth weight (low, ≤ 2.5 kg, versus normal, > 2.5 kg), the level of post-donation albuminuria was significantly higher in the lower birth weight group. Post-donation estimated GFR in the lower birth weight group dropped more from the pre-donation baseline and showed lesser potential to recover. A key study finding is that morphometric measures of the remaining kidney were indistinguishable between the two groups; only the calculated nephron number, based on birth weight, differed. This finding suggests that existing clinical algorithms, which do not incorporate birth weight, do not identify these at-risk donors, who likely account for a fraction of the elevated ESKD risk observed in the kidney donor pool. Notably, several studies have shown higher rates of hypertension, obesity, and diabetes mellitus in adults born with low birth weight [34]. Thus, even without considering nephron endowment, individuals with low birth weights may appear healthy as young adults, but may develop the “second hit” conditions later in life, placing them at risk for progression loss of kidney function post-donation. With the expansion of research efforts in kidney-related precision health, we anticipate more candidate “first hits*”* to be identified, which may be incorporated into donor evaluation and follow-up.

## Where Do We Go From Here? Direction for Future Investigation

Based on the physiologic model proposed above, we make the following recommendations for future research efforts:

1. Refine a framework for individualized donor risk stratification. As the risk of post-donation kidney disease is probably concentrated in high-risk subsets, the logical next step is to refine our detection of that subset. A risk calculator enabling the projection of lifetime ESKD risk in the absence of donation has recently been proposed [33]. A second risk calculator enables the estimate of ESRD risk after donation based on all living donors in the US based on donor age, sex, race, body mass index, and relationship to donor [35]. These calculators are population-based and, owing to the quality of input data, have limited granularity. While providing an estimate of lifetime ESKD risk, these current calculators may fall short in predicting the risk of progressive CKD in individual donor candidates. Incorporating additional information on life expectancy and the expected trajectory of kidney function, including the development of advanced, non-dialysis-requiring CKD, may help to refine clinical decision-making. Surrogates for nephron endowment, to the extent that such information is available, should be routinely incorporated into the database for risk stratification.
2. Establish the clinical significance of the subtle physiologic extra-renal alterations, including cardiac remodeling and asymptomatic aberrations of mineral metabolism, to guide the future medical management of donors.
3. Define blood pressure goals and first-line treatment for hypertension post-donation: As discussed above, hypertension is common after donation and subclinical structural cardiac changes may ensue. Data from the Systolic Blood Pressure Intervention Trial (SPRINT) would suggest that older persons with hypertension and either heightened cardiovascular risk or modest decrements in estimated GFR experience lower rates of death and major cardiovascular events when aiming for a systolic blood pressure target below 120 mmHg [36]. It is reasonable to extrapolate these results to the kidney donor population, particularly those with albuminuria and/or other cardiovascular risk factors. We should formally the test the hypothesis that inhibitors of the renin-angiotensin-aldosterone system (RAAS), through effects on glomerular pressure and cardiac remodeling, are preferable to other antihypertensive classes for the treatment of post-donation hypertension. It may also be relevant to examine the effects of RAAS inhibition in donors, aiming to preserve kidney function by mitigating glomerular hypertension, even in the absence of elevated systemic blood pressures.
4. Standardize usage of International Classification of Diagnosis (ICD) codes to describe the post-donation state. The tenth edition of the ICD (ICD-10) contains a specific code for kidney donor, Z52.4. Standardizing usage of Z52.4 for all kidney donors allows for case identification in future research relying on administrative data.

## Where Do We Go from Here? Practical Recommendation for Care and Follow-Up of Donors

Systematic living donor follow-up is crucial for the practice of living donation for two main reasons. First reason is to provide the appropriate medical care to all living donors, in order to promote physical and psychosocial well-being and to prevent and manage individual clinical problems through surveillance. Second, is to collect accurate information on donor outcomes that can then be used to improve the donor evaluation and counseling process and to provide program-specific feedback to transplant programs.

In keeping with the multiple-hit hypothesis advanced here, we recommend that:

1. The decision to proceed with living kidney donation should best utilize shared decision-making based on individualized donor risk, incorporating new knowledge such “first hits,” as the presence of low birth weight or high-risk APOL1 genotype. All living donors should be queried about birth weight and prematurity, and individuals with documented low birth weight should be regarded as “at risk” for post-donation progressive loss of kidney function. It remains to be seen if all prospective African-American donors must test for APOL1 high-risk alleles, but it may be reasonable to pursue such investigations in the presence of known low-birth weight or prematurity.
2. All living donors should receive long-term clinical care post-donation that focuses on the detection, prevention and management of conditions that may constitute a “second hit,” including arterial hypertension, diabetes mellitus, and obesity. The 2017 Kidney Disease Improving Global Outcomes guidelines [37] recommend annual monitoring and increasing intensity if problems arise, or if the magnitude and persistence of GFR decline or new-onset albuminuria qualifies patient for CKD. As many young donors are at the highest risk of loss to follow-up to the medical system, transplant centers should use the living donation as an opportunity to encourage a continuing relationship between living donors and the medical system and educate donors on the critical importance of lifelong health maintenance and disease prevention.

In the collection of donor outcomes, our perspective draws attention to the decades-long timeline over which post-donation kidney disease may arise. This timeline stresses the importance of maintaining long-term living donor registries, beyond the two-year registry currently mandated by United Network for Organ Sharing in the US. Furthermore, the registry will ideally collect information on intermediate outcomes (i.e., albuminuria) and “second hit” conditions that modulate the course of GFR decline after donation, rather than just terminal endpoints such as death or ESKD.

## Conclusions

In summary, kidney donation is associated with subtle physiologic alterations that do not qualify it as a unique disease in most donors. However, donor subsets at higher risk exist, either due to a “first hit” (in utero or genetic predisposition) present at birth or to the subsequent acquisition of a “second hit” (e.g., hypertension, diabetes mellitus, obesity) later in life. Even then, post-donation kidney disease typically takes decades to manifest and progress. Efforts should focus on individualizing risk prediction prior to kidney donation, preventing acquisition of “second hits” after kidney donation, and establishing a long-term registry to refine the understanding of risk after donation.

## Abbreviations

CKD: Chronic kidney disease
ESKD: End-stage kidney disease
GFR: Glomerular filtration rate
KDIGO: Kidney Disease Improving Global Outcomes
RAAS: Renin-angiotensin-aldosterone system
US: United States
